# Impact of COVID-19 on breast cancer patients and services in a UK region: Protocol for a mixed methods study

**DOI:** 10.1371/journal.pone.0333288

**Published:** 2025-09-29

**Authors:** Helen Julia Mitchell, Charlene M. McShane, Sinéad Teresa Hawkins, Lynne Lohfeld, Paula Darragh, Gareth Irwin, Nicole Lowans, Ann McBrien, Elisabeth Moss, Siobhan O'Neill, Jamie Roebuck, Shreya Sengupta, Meenakshi Sharma, Anna T. Gavin, Damien Bennett

**Affiliations:** 1 Northern Ireland Cancer Registry, Centre for Public Health, School of Medicine, Dentistry & Biomedical Sciences, Queen’s University Belfast, Belfast, Northern Ireland, United Kingdom; 2 Centre for Public Health, School of Medicine, Dentistry & Biomedical Sciences, Queen’s University Belfast, Belfast, Northern Ireland, United Kingdom; 3 Clinical Data Group, Belfast Health and Social Care Trust, Belfast, Northern Ireland, United Kingdom; 4 Patient Advocate, Belfast; Mayo Clinic Rochester, UNITED STATES OF AMERICA

## Abstract

**Background:**

Globally, breast cancer screening, diagnosis and treatment services were significantly impacted by the COVID-19 pandemic. Locally, the Northern Ireland Cancer Registry (NICR) reported an 11% reduction in breast cancers diagnosed April-December 2020 compared with the same period 2018−2019 despite an expected increase. This study aims to identify how the patient journey was impacted by the COVID-19 pandemic and how service provision can be improved.

**Methods:**

Population-based quantitative data will be collected by NICR for individuals diagnosed with invasive breast cancer (ICD 10 code C50). Data on presentation, investigations, comorbidities (including COVID-19 infection), molecular markers, stage at diagnosis, treatment, and survival will be examined. Two time periods will be compared: March-December 2018 (pre-COVID-19 cohort) and March-December 2020 (COVID-19 cohort). Analysis will include descriptive statistics, chi-square tests and ANOVAs. Patient experiences of living with breast cancer and interacting with the healthcare system during the pandemic (March 2020-April 2024) will be captured through an online, self-completed, anonymous cross-sectional international survey. Quality of life will be captured in the survey using the EQ-5D-3L. Detailed descriptive qualitative interviews will be undertaken to better understand the lived experience of breast cancer and recommend ways that services and support programs can effectively meet the needs of this patient group. This study benefits from the inclusion of patient representatives who have been involved since the inception of this project.

**Discussion:**

This study aims to quantify the impact of the COVID-19 pandemic on breast cancer patient outcomes in a UK region and make recommendations to improve patient care and outcomes in the short and long term**.**

## Background

During 2020 the health service in Northern Ireland (NI), as elsewhere, experienced a surge in COVID-19 patients resulting in significant disruptions to non-COVID-19 health and social care (HSC) services including cancer care [1,2]. Ensuring optimal outcomes for cancer patients requires timely diagnosis and prompt treatment [3]. However, COVID-19 pandemic related disruptions such as suspension of cancer screening, stricter guidelines on accepting referrals from general practitioners and capacity restrictions on diagnostic and treatment interventions have raised concerns about delays to diagnosis, especially for those requiring time-sensitive care [4,5]. Breast cancer (BC) is the most common cancer in UK women [6], and early-stage BC usually has excellent prognosis with current treatments [7]. However, delays to presentation can mean BC is diagnosed at a later stage, leading to reduced quality-of-life and survival outcomes with an associated increase in the cost of care [8]. In NI population-based BC screening is offered to women aged 50–70 years every three years. Overall levels of breast screening uptake in NI are high (77% in 2019 [9]). Between 2012 and 2016, 30% of BC patients in NI were diagnosed through screening [10]. Furthermore, BC patients of screening age tend to be diagnosed at an earlier stage (48% aged 50–70 stage 1 vs. 40% all ages) [10]. There is a marked difference in survival between early and late stage BC, with 97% five-year survival for stage 1 disease but only 20.2% for stage 4 disease for patients diagnosed 2012–2016 [11].

NICR is a population-based cancer registry (PBCR) covering 1.91 million people providing high quality cancer data [12]. Since March 2020, NICR has monitored the impact of the COVID-19 pandemic on cancers in NI by tracking the number of pathologically diagnosed cancers [13]. For BC, a significant reduction of 34% was reported in pathologically diagnosed cases during the first three months of the pandemic (April-June 2020) [14].

During the initial COVID-19 pandemic period, treatment protocols were altered and access to operating theatres was limited [15]. The use of preoperative or ‘bridging’ hormone therapy as first treatment became more commonplace than before the COVID-19 pandemic [16]. This meant that even for patients meeting the 62-day cancer waiting time to begin treatment, initiation with hormone therapy rather than the normal treatment pathway of surgery, radiotherapy or chemotherapy [17]. The Association of Breast Surgery (ABS) published their recommendations on 15^th^ March 2020 which suggested that that immediate breast reconstruction be delayed to a later time [18]. Some Health and Social Care Trusts (HSCTs) in NI followed ABS’ prioritisation guidance [19]). However others, depending on capacity issues, followed the Federation of Surgical Speciality Associations (FSSA) guidance, with all Trusts eventually moving to follow FSSA guidance [20]. In addition, private sector operating theatres were utilised as low risk COVID-19 sites for surgery. Other changes in treatment pathways include the regional move to hypo-fractionated breast radiotherapy protocols [21], where patients received a higher dose of radiation over five days instead of the conventional lower dose over 15 days and changes in chemotherapy delivery to balance the benefits of chemotherapy with the possible risk of COVID-19 infection whilst receiving chemotherapy [22].

The impact of these sudden and substantial changes to BC diagnostic, treatment and follow-up pathways is not yet known. Using quantitative and qualitative data from a PBCR, patient clinical data, an online patient survey and in-depth patient interviews, this project aims to examine the impact of COVID-19 pandemic on BC patients in NI and make recommendations to improve patient care and outcomes.

## Methods/design

### STUDY 1: Establishing population-based NI breast cancer cohort (PRE-COVID-19 cohort vs COVID-19 cohort)

#### Aim.

Investigate the extent to which the initial COVID-19 pandemic impacted BC patient care.

#### Objectives.

i. To describe in detail routes to diagnosis (screening/emergency/other), investigations, stage at presentation, treatments received and survival for BC patients diagnosed in 2018 (PRE-COVID-19 cohort) compared to those used for patients diagnosed in 2020 (COVID-19 cohort).
ii. To examine differences in care between the two cohorts taking account of patient age, socioeconomic group, and comorbidities.
iii. To determine the number of BC patients admitted to hospital with COVID-19, their route to diagnosis and outcomes.
iv. To measure how closely the investigations and treatments received match NICE COVID-19 guidance [7].

#### Characteristics of study population.

For this element of the study, patients with invasive breast cancer (ICD10 code C50), aged 18 years and older who were diagnosed between 1^st^ March and 31^st^ December 2018 (pre-COVID-19 cohort) or 1^st^ March – 31^st^ December 2020 (COVID-19 cohort) and residing in NI will be included. The 2018 time period has been selected to avoid including patients newly diagnosed in 2019 but who may still have been receiving treatment in 2020.

#### Exclusion criteria.

Individuals with cancer of unknown primary site, metastasis in the breast originating from another primary site, carcinoma in situ, non-invasive tumours and dysplasia will be excluded from the current study. BC patients will also be excluded if their records lack sufficient information (e.g., death certificate only registration). However, we do not anticipate this being a significant problem as the most recent NICR breast cancer audit (in 2012) found that patient records had a 99.4% data completeness rate [23]. Patients under the age of 18 years old at the time of their cancer diagnosis were also excluded.

#### Data collection.

Highly trained NICR Cancer Intelligence Officers (CIOs) will extract and quality check data on BC patients diagnosed during the timeframes of interest. All data extraction, quality control and analysis will take place in the secure (ISO 27001 accredited) NICR environment. The NICR has data-sharing agreements (including with the private healthcare sector) and ethical approvals to receive cancer patient data from numerous clinical sources across NI.

**Fig 1:**
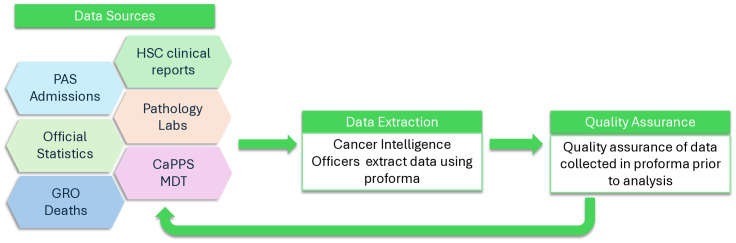
Data collection.

Data will be obtained from HSC clinical systems reports and the Patient Administration System (PAS), which provides demographic information such as patient age, pre-existing comorbidities resulting in hospital admission and postcode of residence (enabling deprivation and rurality to be determined). These data will be supplemented by electronic downloads from cytopathology and histopathology laboratories which provide information on tumour morphology, immunohistochemical and molecular markers, allowing the sub-categorisation of BC type. The clinical oncology system, HSC Trust staff input and data from the MDT tracking system (CaPPS) will provide treatment data, supplementary information for staging and help explain delays, if they exist. Date of death and cause of death will also be included (Fig 1).

Healthcare staff at collaborating HSC Trusts across NI will provide additional data and clinical insights not available to NICR (e.g., reasons for delays and details of treatment changes). Some cancer treatments moved to the private sector as a result of the COVID-19 pandemic. NICR has access to this data but will also link with local clinicians to confirm the accuracy of this information.

Data collection has been completed for the quantitative part of the study and analysis is underway. It is anticipated that analysis will be completed in September 2025.

#### Data analysis.

Quantitative analysis of NICR data will include information on method of presentation (screening/ emergency/other), investigations undertaken, BC subtype, patient comorbidities (including COVID-19 infection), stage at diagnosis, and treatment received with associated timings (e.g., time from diagnosis to first treatment, etc.) for BC patients diagnosed in NI during the study periods. The hypotheses in Table 1 will be tested by comparing data from the pre-COVID-19 and COVID-19 cohorts. Death record data will be used to assess survival differences using Kaplan-Meier curves, with more detailed Cox proportional hazards analysis used where appropriate, adjusting for key confounders. Directed acyclic graphs (DAGs) will be drawn to aid identification of potential variables to include in models.

**Table 1:** Hypotheses to be tested and historic data.

Hypothesis/Questions to be answered by comparison of pre-COVID-19 with COVID-19 era patients	Information below is the average for BC patients diagnosed 2013–2017 in Northern Ireland
**Will age at diagnosis increase due to** i) **Aging of the population?** ii) **Loss of screen detected cancers?**	44.8% of women over 65 19.4% diagnosed under 50 Median age at diagnosis = 62 years old
**Will stage at diagnosis increase with less early stage I and more late stage IV?**	39.8% diagnosed at Stage 1 5.1% diagnosed at Stage 4
**Will proportion of patients presenting as emergency change, e.g., initially decrease but later increase?**	Emergency presentation 2012–2016 3.7% least deprived 4.8% most deprived 29.1% presented via screening 4.1% via A&E^20^
**How have treatment schedules changed?**	
**Has survival been affected?**	Patients diagnosed 2012–2016 – One year observed survival estimates 94.7% (77.2% for 5-Year survival)
**How has the COVID-19 pandemic affected different socioeconomic groups?**	(2012–2016 Patients) Breast Cancer detected via screenings: 31.9% least deprived and 25.8% more deprived No socioeconomic difference noted in the levels of breast cancer presenting (2013–2018 patients combined)

Descriptive statistics (frequencies and proportions over time) will be presented for both cohorts, along with comparisons on a month-by-month basis. Tests for significant differences in demographic and cancer variables between the two time periods will be done using chi-squared tests and ANOVAs. Subgroup analyses will be conducted by age and socioeconomic group, rurality and stage at diagnosis using multivariate logistic regression. The information on male breast cancers will be descriptive only as the sample size is small.

### STUDY 2: Understanding *the* BC patient experience using *a* mixed-methods approach

This aspect of the study comprises two elements: [1] an online cross-sectional survey and [2] in-depth qualitative interviews.

#### Aims.

Develop an understanding of the lived experience of BC patients during COVID-19 and the extent to which the COVID-19 pandemic affected BC services from the patient perspective.

#### Objective.

To document the psychosocial impact of the COVID-19 pandemic on BC patients, both newly diagnosed and prevalent (existing) cases, via a cross-sectional online international survey, and explore BC patients’ experiences and recommendations for care through individual in-depth interviews.

#### Study participants.

*Survey:* Individuals, irrespective of gender, self-reporting a BC diagnosis from any country and at any timepoint, aged 18 or older, were eligible to take part in an anonymous, online survey. Only individuals who agreed to consent to participate will be included the analysis. The survey will be offered in English language only.

*Qualitative interviews with BC patients in NI:* At the end of the anonymous survey, participants will be given the option of indicating interest in being interviewed about their experiences of living with BC during the pandemic. They can provide their name and contact information, which is delinked from the survey. Approximately 20 women and a small number of men who provide contact information at the end of the survey will be recruited to take part in individual in-depth interviews with a member of the study team. Up to four additional interviews may be conducted if data saturation is not reached. Additional recruitment may take place if required (e.g., advertising the study via charities, local patient advocacy groups etc.). Participants will be required to renew their previous consent to participate in the study prior to the interview. All interviews will take place online and in English.

#### Data collection.

*Survey:* A bespoke survey has been developed by the study team following a scoping review of the literature and in consultation with key stakeholders, including BC patients and breast cancer care providers in NI [24]. The survey comprises a series of closed- and open-ended questions to explore the BC patient experience of interacting with the healthcare systems during the COVID-19 pandemic (for example, consultation experience, e.g., telephone and online consultations), healthcare appointments, COVID-19 infection and vaccination history, impact of pandemic on general wellbeing, and suggested improvements for BC services. The EuroQoL EQ-5D-3L has been included to ascertain Quality of Life. Additional questions have been included to capture information on participant demographics and BC diagnosis (e.g., time of diagnosis, stage at diagnosis, recurrence status). The survey was reviewed and pre-tested by BC patients and experts before being launched using the Smart Survey platform in September 2023 and closed in April 2024. *S*ocial media, including paid Facebook advertisements, as well as local cancer charities and newspapers/media outlets were used to advertise the survey. To increase the number of participants from NI, posters were distributed for display in the local community on noticeboards and in shop windows. Analysis of the survey data is underway and it is anticipated that results will be available in September 2025.

*Interviews:* A descriptive qualitative study will be done in which approximately 20 women and up to five men with BC living in NI will take part. Based on methodological guidelines, this number of interviews should provide enough data to reach saturation, or the point where no new information emerges from the last data collection, and all categories or themes the researchers identify are well understood and fully supported by quotes from the interviews [25]. Data saturation is often used to determine sample size in qualitative research. However, guidelines for the Descriptive Qualitative approach [26,27], note that approximately 20 participants with relevant knowledge and experience or “information power” [28]will provide data with sufficient breadth and depth to answer the research question. An additional way to reach data saturation will be for the investigator to ask each participant to comment on a summary of statements they make on each topic during the interview. Given that participants will have first responded to an online survey, it is likely that most people will choose to be interviewed online via Microsoft Teams. However, to ensure we do not exclude patients based on their access to technology, participants can instead choose to complete the interview by phone or in person. To protect participant confidentiality, each participant in the interview portion of the study will be assigned a unique identification code at recruitment. Contact information (names and email addresses) will be stored separately in a secure, password-protected file, accessible only to the research team. This list will be used solely for communication and to link participants to their interview transcripts, which will be pseudonymised prior to analysis by removing all direct identifiers and replacing them with ID codes. Audio files made during interviews, to produce accurate transcripts, will be deleted following transcript verification. The linkage key will be destroyed at the end of the analysis, after which the transcripts will become fully anonymised. This approach is consistent with data protection principles outlined in the UK GDPR and is widely endorsed in qualitative research practice as an ethical strategy for managing re-identifiable data while minimising risk [29]. Recruitment for the interviews is anticipated to be completed in August 2025, however if data saturation is not complete at this time then further recruitment will be carried out. It is anticipated that results for the interviews will be available in October 2025.

#### Data analysis.

*Survey:* The data will be extracted from the Smart Survey platform and analysed using STATA or alternative statistical software programme. Descriptive and inferential statistics will be used to describe the data. The EQ-5D-3L questionnaire results will be analysed according to published guidelines and findings compared to population norms. Regression analyses adjusting for potential confounders will also be conducted. Open-ended responses to the survey will be analysed using the Qualitative Content Analysis method [30] in which analysts enter all responses to each question onto an Excel spreadsheet, one answer per row. They then write a brief description of each statement’s meaning and use this to develop labels (codes + subcodes). Then the codes/subcodes will be clustered into categories that reduce the data to a core set of concepts. Major themes will be identified as those present in at least half of all responses to a question. All findings will be supported with anonymised quotes from respondents.

*Interviews:* Audio files from the interviews will be used to create verbatim anonymous transcripts that will be analysed following the steps of qualitative content analysis [30]. We will strive for code and theme saturation through a stepwise analysis process. Each member of the analysis team will individually code the first three transcripts and reach agreement on the initial analysis framework. All codes must have sufficient evidence (data) to be included in the framework. The process will continue until there are few or no changes and the analysts will finish coding the transcripts, noting any changes or new insights they develop. Discussion will then focus on generating themes from clusters of codes. We will determine if we reach saturation by systematically recording changes to the framework in a codebook listing all codes and subcodes along with a brief definition and exemplary quote, highlighting changes identified in team meetings. We will also produce a retrospective saturation map in which we systematically record when each code and theme first appeared, where redundancy occurs, when no new codes or themes emerge along with new ones from later transcripts [25,31,32]. The results will be discussed and agreed to by the wider research team, including the PPI representatives. Although we recognise the importance of including insights from men on how COVID affected their breast cancer journeys, the low prevalence means we may not recruit any men for interviews. If that occurs, we will note this as a limitation of our study and highlight this as an important area for future research.

### Mixed -methods integration

We intend to integrate the components of this study as far as possible. One opportunity would be to explore the impact of delayed reconstruction on both clinical and patient reported outcomes. To do this we will review the quantitative data on the number of delayed procedures and factors such as post-operative complications and survival and then review the survey and qualitative interview transcripts. In doing so we hope to integrate the clinical data with the real-world impact of changes to surgical procedures and capture the patient voice.

## Ethical approval and consent to participate

### Ethical approval

NICR has ethical approval from the Office for Research Ethics Committees of Northern Ireland (ORECNI) (Ref: 20/NI/0132, IRAS project ID: 288121), for the collection and use of routinely collected data, relating to cancer patients, within the fields of health and social care research. This ethical approval covers the quantitative methods of this study.

Ethical approval for the survey and interviews has been granted by the Faculty of Medicine, Health and Life Sciences at Queen’s University Belfast (MHLS 22_133). Informed written consent was collected prior to completion of the survey as part of the information sheet and was documented as part of the survey response. At the end of the survey participants were requested to give informed consent if they were happy to be contacted to take part in the interviews. Written informed consent was collected prior to the interviews taking place.

### Consent for publication

Informed consent is not required for the publication of the data collected in the quantitative aspect of the study. Informed consent has been collected for publication of the results of the survey and the interviews.

### Patient and public involvement

Patient and Public Involvement (PPI) is incorporated throughout this project, with patient advocates involved in the initial concept and named on the funding application. BC patients and clinical staff are active members of both the implementation and project steering groups. Both patients and clinical experts have been involved in the development of the survey questions, survey piloting, and co-design and piloting of the interview guide. This led to the inclusion of questions/topics in the survey that were not previously prioritised. Preliminary findings will be presented to PPI representatives to support interpretation of the findings. PPI representatives will also be named co-authors on all publications and involved in the review process.

### Data security and information governance

The BC cohort data will be held within the secure NICR (ISO 27001 accredited) environment. The survey and qualitative data will be stored on secure password protected Queen’s University Belfast computers/servers. Responses to the survey or interviews will not be linked to cancer data held by NICR.

## Discussion

This study, by including the patient voice, should provide an innovative perspective on the impact of the COVID-19 pandemic on cancer patients. The inclusion of patient representatives from the start of the study has provided a basis for ensuring the patient voice is portrayed.

### Strengths and Limitations

This study benefits from using high-quality population-based data linked to hospital administrative data, as well as data collected by clinical staff. The aim is to provide an accurate picture of changes to BC treatment pathways, such as surgery, during the COVID-19 pandemic. Another strength of this study is that it considers the impact of COVID-19 on breast cancer from a wide variety of perspectives (e.g., PBCR data, clinical data on treatment changes, patient outcomes and experience, etc.), which is vital to understand the far-reaching impact of the pandemic. The findings of this work will help inform service provision should another pandemic or service disruption occur.

A limitation of this study is that quantitative data were collected retrospectively. Therefore, changes in patient outcomes may be due to unmeasured confounders. Secondly, the initial methodology of gathering survey responses using the researchers’ own networks and NICR social media accounts stagnated at around 200 responses for a period of two months. In order to address this issue, an alternative strategy was adopted including the use of paid Facebook advertisement and posters. The survey opening was also extended to increase responses.

## Abbreviations

ABS: Association of Breast Surgery
BC: Breast Cancer
FSSA: Federation of Surgical Specialty Associations
HSC: Health and Social care
ISO: International Organization for Standardization
MDT: Multi-Disciplinary Team
NI: Northern Ireland
NICE: National Institute for Health and Care Excellence
NICR: Northern Ireland Cancer Registry
PAS: Patient Administration System
PBCR: Population Based Cancer Registry
PPI: Patient and Public Involvement
RoI: Republic of Ireland
